# Use of the dye-guided sentinel lymph node biopsy method alone for breast cancer metastasis to avoid unnecessary axillary lymph node dissection

**DOI:** 10.3892/etm.2013.1445

**Published:** 2013-12-11

**Authors:** TOMOKO TAKAMARU, GORO KUTOMI, FUKINO SATOMI, HIROAKI SHIMA, KEISUKE OHNO, HIDEKAZU KAMESHIMA, YASUYO SUZUKI, TOUSEI OHMURA, HIROYUKI TAKAMARU, MASANORI NOJIMA, MITSURU MORI, KOICHI HIRATA

**Affiliations:** 1 First Department of Surgery, Sapporo Medical University, Sapporo, Hokkaido 060-8543, Japan; 2 First Department of Internal Medicine, Sapporo Medical University, Sapporo, Hokkaido 060-8543, Japan; 3 Department of Public Health, Sapporo Medical University, Sapporo, Hokkaido 060-8556, Japan

**Keywords:** axillary lymph node dissection, breast cancer, dye-guided method, sentinel lymph node biopsy, prediction of lymph node metastasis

## Abstract

For sentinel lymph node biopsy (SLNB), a combination of dye-guided and γ-probe-guided methods is the most commonly used technique. However, the number of institutes in which the γ-probe-guided method is able to be performed is limited, since special equipment is required for the method. In this study, SLNB with the dye-guided method alone was evaluated, and the clinicopathological characteristics were analyzed to identify any factors that were predictive of whether the follow-up axillary lymph node dissection (ALND) was able to be omitted. A total of 374 patients who underwent SLNB between 1999 and 2009 were studied. The SLN identification rate was analyzed, in addition to the false-positive and false-negative rates and the correlation between the clinicopathological characteristics and axillary lymph node metastases. The SLN was identified in 96.8% of cases, and, out of the patients who had SLN metastasis, 63.0% did not exhibit metastasis elsewhere. The sensitivity was 96.4% and the specificity was 100%. The false-negative rate was 3.6%. Univariate analyses revealed significant differences in the lymph vessel invasion (ly) status, nuclear grade (NG), maximum tumor size and the percentage of the area occupied by the tumor cells in the SLN (SLN occupation ratio) between the patients with and without non-SLN metastasis, indicating that these factors may be predictive of axillary lymph node metastasis. Multivariate analysis revealed that ly status was an independent risk factor for non-SLN metastasis. In conclusion, SLN with the dye-guided method alone provided a high detection rate. The study identified a predictive factor for axillary lymph node metastasis that may improve the patients’ quality of life.

## Introduction

Axillary lymph node dissection (ALND) provides information for staging and prognosis that may be used to design a therapeutic strategy (1–3). As a result of this, a large number of patients with breast cancer in the past underwent routine ALND; however, in many of the patients, the cancer was revealed to be node-negative, and surgery unnecessarily exposed them to perioperative risks and increased long-term morbidity (4). Therefore, sentinel lymph node biopsy (SLNB) has widely replaced conventional ALND as a routine axillary staging method in breast cancer surgery. The SLNB procedure is accurate and safe (5–8) and results in substantially less postoperative morbidity than ALND (9,10).

Dye-guided and γ-probe-guided methods, separately and in combination, are used for SLNB. The dye-guided method alone for breast cancer is considered to be inferior to the γ-probe-guided and combined methods in terms of its accuracy and false-negative rate. Cox *et al*(11) revealed the identification rates for SLNB to be 80.3% for the dye-guided technique, 88.6% for the γ-probe-guided method and 96.7% for the combination, based on a large number of procedures performed in a single center. Kim *et al*(12) showed that the rates for successfully identifying the SLN were 83.1, 89.2 and 91.9%, for the dye-guided-alone, γ-probe-guided-alone and combination methods, respectively [with the combination method being significantly more efficacious (P=0.007)], while the false-negative rates were 10.9, 8.8 and 7.0%, respectively (P=0.047).

The dye-guided method has the significant advantages of being more convenient to perform, less costly and possible to conduct in any institute with no special facilities. Although it requires a certain training period, this method may be preferable for all clinicians if its accuracy is demonstrated to be comparable to that of the combination method. Morrow *et al*(13) reported that there were no significant differences between the procedures with regard to the identification rate, accuracy, time spent for identification and number of SLNs. In the present study, the success rate in identifying the SLN, the accuracy and the SLN occupation ratio using the dye-guided method alone were investigated. Furthermore, the possibility of obviating the requirement for ALND in SLNB-positive cases was explored.

## Materials and methods

### Patients

From January 1999 to December 2009, 374 patients with primary breast cancer underwent SLNB using the dye-guided method alone followed by radical surgery at Sapporo Medical University Hospital (Sapporo, Japan). The medical records of these patients were analyzed retrospectively and their clinicopathological characteristics were evaluated. This study was approved by The Ethics Committee of Sapporo Medical University (Sapporo, Japan). Informed consent was obtained from all patients prior to enrollment.

### Surgical procedure

Under general anesthesia, 2–4 ml blue dye (indigo carmine) was injected at two sites surrounding the primary tumor and areola. Approximately 5 min later, a blunt dissection was performed through the mastectomy incision or a separate axillary incision until a dye-stained lymphatic tract or node was identified.

### Pathological assessment

The SLN was split into three to four sections and analyzed as frozen specimens during surgery. These specimens were fixed in 10% formalin, stained with hematoxylin and eosin (H&E) and then re-examined histologically following surgery. The SLN area and the area of tumor cells in the SLN were calculated by multiplying the longest diameter by the minor axis. The SLN occupation ratio was defined as the proportion of area that was occupied by tumor cells to the total SLN area. As a feasibility study, ALND was completed for cases of at least level I or II following SLN excision in the first 54 cases to undergo surgery.

### Statistical analysis

Continuous variables (age, maximum tumor size and SLN occupation ratio), dichotomous variables (estrogen receptor, progesterone receptor and human epidermal growth factor receptor type 2 status) and categorical variables [lymph vessel invasion (ly) status, vessel invasion (v) status and nuclear grade (NG)] were analyzed in a univariate logistic regression model using either the t-test or the χ^2^ test. All variables were subsequently analyzed in a multivariable regression model. A receiver operating characteristic (ROC) curve was drawn on the basis of the SLN occupation ratio, and the area under the curve (AUC) was calculated using SPSS version 11.5 statistical software (SPSS, Inc., Chicago, IL, USA). GraphPad Prism version 5.0.2 (GraphPad Software, La Jolla, CA, USA) was used for all analyses. P<0.05 was considered to indicate a statistically significant difference.

## Results

**Figure 1:**
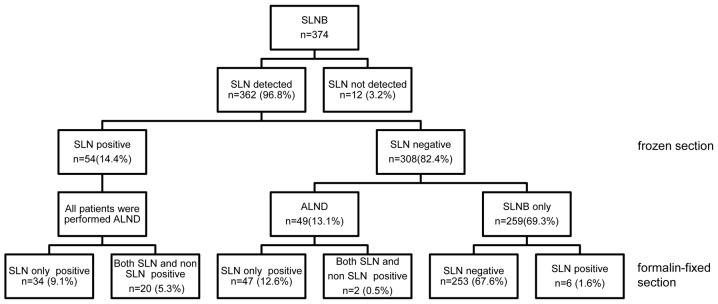
Patient status. SLNB, sentinel lymph node biopsy; SLN, sentinel lymph node; ALND, axillary lymph node dissection.

The clinicopathological characteristics of the patients are shown in Table I. The SLN was successfully identified in 362 of the 374 patients (96.8%). The average number of resected lymph nodes was 1.9 (range, 1–10). A total of 54 patients were identified to have SLN metastases and ALND was performed in these patients. In 34 of the 54 patients (63.0%) with axillary metastases, metastatic nodes were only observed in the SLN. In the remaining 20 patients (37.0%), metastases were observed in the SLN and the non-SLNs. Of the 308 patients without SLN metastases in the frozen sections, 49 underwent ALND. Two of these 49 had positive axillary lymph nodes in the formalin-fixed sections. Among the 259 patients who did not undergo ALND, six had positive formalin-fixed sections (Fig. 1).

### Postoperative diagnosis of SLN metastases

The sensitivity for the identification of a histologically positive node was 96.4% (54/56) (Table IIA), and the specificity was 100%. The accuracy of SLN biopsy for the detection of metastatic disease was 98.1% (101/103). The positive predictive value was 100% (54/54) and the negative predictive value (the correlation between the negative SLNs and the negative axillary nodes) was 95.9% (47/49), with a false-negative rate of 3.6% (2/56).

### Intraoperative diagnosis of SLN metastases

In 362 patients, the intraoperative diagnosis was confirmed by the final histological examination (with formalin-fixed specimens). However, in eight patients (12.9%) there was a false-negative intraoperative diagnosis and a tumor was identified on a permanent section of either the SLN or the non-SLNs. As a result, a diagnostic accuracy of 97.8%, a sensitivity of 87.1% and a specificity of 100% were achieved with H&E staining of the frozen sections (Table IIB).

### Clinicopathological factors correlated with non-SLN metastasis

In 63% of the SLN-positive cases for which ALND was performed, metastasis was limited to the SLN. This result suggested the possibility that the resection of SLNs only may be sufficient to treat in excess of half of the cases with positive SLNs. Therefore, the cases were analyzed to reveal which factors were likely to indicate the potential for omitting the following ALND.

To investigate which factors correlated with non-SLN metastasis, the clinicopathological characteristics of the patients who underwent ALND and had SLN and non-SLN metastases were compared with those who underwent ALND and just had SLN metastasis. Univariate analyses revealed significant differences in the ly status (P=0.0002), NG (P=0.0433), maximum tumor size (P=0.0317) and the SLN occupation rate (P=0.0017) between the two groups. The results of the analyses for various characteristics are summarized in Table III.

Multivariate analysis revealed that ly status was an independent risk factor for non-SLN metastasis (Table IV). An ROC curve was generated to assess the clinical utility of the SLN occupation ratio for the prediction of non-SLN metastasis (sensitivity, 70.0%; specificity, 61.8%; AUC, 0.736).

### Axillary recurrences

In this study group, 5 out of the total 374 cases (1.3%) had an axillary recurrence. Out of all the patients, 259 patients received SLNB alone and 4/259 patients (1.5%) showed axillary recurrence. By contrast, patients who received ALND had no axillary recurrence. The SLN was not able to be detected in 12 cases, and axillary recurrence was observed in one of these cases.

## Discussion

In the present study, the SLN identification rate with the dye-guided method alone was 97%, which was comparable with previous studies (11,12). The population of patients who had SLN metastasis was also consistent with those of previous studies. These data indicated that the dye-guided method alone was sufficient to detect SLN metastasis. In addition, the dye-guided method requires no special equipment, so widespread application may be expected in any institute.

In SLN metastasis-positive cases, subsequent ALND is generally performed. However, in the group observed in the present study, 63% of the patients had no axillary metastasis. These results may indicate that in excess of half the patients receive unnecessary additional surgery. In the management of breast cancer, ALND may dam up the flow of lymph vessels and result in lymphedema in the patient’s arm. Although breast cancer is a malignant disease, survival may be relatively long subsequent to the first surgical therapy, so it is important to support the patient’s quality of life (QOL).

The possibility of predicting axillary lymph node metastasis by the analysis of the SLN status was investigated. The aim of identifying the factors that predict axillary metastasis was to contribute to avoiding unnecessary ALND. Univariate analysis revealed significant differences in ly status, NG, maximum tumor size and the SLN occupation ratio between patients with axillary metastasis and those with SLN metastasis only. In breast cancer, lymph node metastasis may spread through the lymph nodes stepwise, following the lymph vessel route. Thus, ly status, NG, tumor size and the SLN occupation ratio are associated with axillary metastasis, and the analysis of these factors is likely to enhance our capability to predict the patients with axillary lymph node metastasis.

The multivariate analysis of the clinicopathological factors, including those mentioned previously, revealed a correlation between axillary metastasis and ly status. However, since it is necessary to assess these factors in permanent formalin-fixed specimens, it is difficult to use them to decide on the surgical procedure during the surgery itself. Therefore, in the present study, the clinical usefulness of the SLN occupation ratio, which is able to be diagnosed during surgery, was investigated. It was demonstrated that the greater the degree of the area occupied by tumor cells, the more invasive the tumor was to lymph vessels, with greater proliferative ability, a larger number of malignant cells, a more metastatic character, and the longer the time after metastasis. Thus, this parameter was expected to be correlated with axillary metastasis. The ROC curve analysis showed sufficient sensitivity and specificity (70.0 and 61.8%, respectively) when the cut-off point for deciding whether it was necessary to perform ALND for patients who may not actually have axillary lymph node metastasis was 12.9%. Thus, a predictive factor for axillary lymph node metastasis in breast cancer was identified, which suggests that it may be possible to avoid unnecessary axillary lymph node resection, and thereby improve the patients’ QOL.

## Figures and Tables

**Table I:** Characteristics of 374 patients who underwent SLNB.

Clinical features	No. of patients (%)
Mean age, years (range)	56.7 (23–87)
Location of tumor	
Medial	103 (27.5)
Lateral	264 (70.6)
Central	7 (1.9)
Clinical tumor stage	
T0	14 (3.7)
T1	175 (46.8)
T2	161 (43.0)
T3	19 (5.1)
T4	5 (1.3)
Clinical nodal stage	
N0	332 (88.8)
N1	41 (11.0)
N2	1 (0.3)
N3	0 (0.0)
No. of SLN	
1	149 (39.8)
2	130 (34.8)
3	56 (15.0)
4	18 (4.8)
≥5	9 (2.4)
Not detected	12 (3.2)

SLNB, sentinel lymph node biopsy.

**Table II:** Diagnosis of sentinel lymph node metastases.

A. Postoperative diagnosis			

Postoperative diagnosis	SLN or non-SLN-positive	SLN and non-SLN-negative	Total
SLN-positive	54	0	54
SLN-negative	2	47	49
Total	56	47	103

B. Intraoperative diagnosis			

Intraoperative diagnosis	SLN-positive	SLN-negative	Total

SLN-positive	54	0	54
SLN-negative	8	300	308
Total	62	300	362

Postoperative diagnosis: The sensitivity for sentinel lymph node (SLN) metastasis was 96.4%, the specificity was 100% and the false-negative rate was 3.6%. Intraoperative diagnosis: A diagnostic accuracy of 97.8%, a sensitivity of 87.1% and specificity of 100% were achieved with hematoxylin and eosin (H&E) staining in the frozen sections; the false-negative rate was 12.9%.

**Table III:** Clinicopathological factors correlated with non-SLN metastasis.

Clinical feature	SLN only	SLN and non-SLN	P-value
Mean age (years)	59.76	59.85	0.9820
Tumor size (cm)	2.34	2.92	0.0317^a^
ly			0.0002^b^
ly0	12	0	
ly1–3	20	20	
v			0.2346
v0	29	15	
v1	3	5	
NG			0.0433^a^
1	20	6	
2–3	11	12	
Unknown	3	2	
ER status			0.7208
Negative	7	3	
Positive	24	17	
Unknown	3	0	
PgR status			
Negative	10	2	0.0949
Positive	21	18	
Unknown	3	0	
HER2 status			0.6958
Negative	27	16	
Positive	4	4	
Unknown	3	0	
SLN occupation ratio (%)	21.47	46.75	0.0017^b^

P-values were calculated using either the t-test or the χ^2^ test.

a P<0.05,

b P<0.01.

SLN, sentinal lymph node; ly, lymph vessel invasion; v, vessel invasion; NG, nuclear grade; ER, estrogen receptor; PgR, progesterone receptor; HER2, human epidermal growth factor type 2; SLN occupation ratio, percentage of SLN area occupied by tumor cells.

**Table IV:** Multivariate analysis.

Risk factor for SLN metastasis	P-value	Relative risk	95% CI
Age	0.0938	1.00	0.93–1.07
Maximum tumor size	0.1477	2.09	0.81–8.24
ly			
ly0	0.0138^a^	1.00	1.95–5.14×10^19^
ly1–3		7.08×10^8^	
v			
v0	0.4950	1.00	0.27–20.47
v1		2.03	
NG			
1	0.1258	1.00	0.68–29.32
2–3		4.05	
ER status			
Negative	0.8965	1.00	0.105–12.33
Positive		1.16	
PgR status			
Negative	0.0823	1.00	0.791–145.56
Positive		7.30	
HER2 status			
Negative	0.2760	1.00	0.43–25.07
Positive		3.00	

a P<0.05.

SLN, sentinel lymph node; CI, confidence interval; ly, lymph vessel invasion; v, vessel invasion; NG, nuclear grade; ER, estrogen receptor; PgR, progesteron receptor; HER2, human epidermal growth factor type 2.
